# Funding Based on Needs? A Study on the Use of Needs Assessment Data by a Major Humanitarian Health Assistance Donor in its Decisions to Allocate Funds

**DOI:** 10.1371/currents.dis.d05f908b179343c8b4705cf44c15dbe9

**Published:** 2014-05-16

**Authors:** Emma Olin, Johan von Schreeb

**Affiliations:** Global Health/IHCAR, Department of Public Health, Karolinska Institute, Stockholm, Sweden; Centre for research on health care in disasters, Department of Public Health, Karolinska Institute, Stockholm, Sweden

## Abstract

Background: International humanitarian assistance is essential for disaster-affected populations, particularly in resource scarce settings. To target such assistance, needs assessments are required. According to internationally endorsed principles, donor governments should provide funding for humanitarian assistance based on need. Aim: The aim of this study is to explore a major donor’s use of needs assessment data in decision-making for allocations of funds for health-related humanitarian assistance contributions. Setting: This is a case study of the Swedish International Development Cooperation Agency (Sida), a major and respected international donor of humanitarian assistance. Methods: To explore Sida’s use of needs assessment data in practice for needs-based allocations, we reviewed all decision documents and assessment memoranda for humanitarian assistance contributions for 2012 using content analysis; this was followed by interviews with key personnel at Sida. Results: Our document analysis found that needs assessment data was not systematically included in Sida’s assessment memoranda and decision documents. In the interviews, we observed various descriptions of the concept of needs assessments, the importance of contextual influences as well as previous collaborations with implementing humanitarian assistance organizations. Our findings indicate that policies guiding funding decisions on humanitarian assistance need to be matched with available needs assessment data and that terminologies and concepts have to be clearly defined. Conclusion: Based on the document analysis and the interviews, it is unclear how well Sida used needs assessment data for decisions to allocate funds. However, although our observations show that needs assessments are seldom used in decision making, Sida’s use of needs assessments has improved compared to a previous study. To improve project funds allocations based on needs assessment data, it will be critical to develop distinct frameworks for allocation distributions based on needs assessment and clear definitions, measurements and interpretations of needs. Key words: Needs assessment, humanitarian assistance, disasters, donor decision-making

## Introduction

A disaster is ”a serious disruption of the functioning of a community or a society involving widespread human, material, economic or environmental losses and impacts, which exceeds the ability of the affected community or society to cope using its own resources” 1. Disasters affect more than 200 million people each year 2. They are also predicted to increase 3 ^,^ 4 ^,^ 5. People and societies in low and middle-income countries are most vulnerable to disasters 2,6. Thus, external humanitarian assistance will be needed to assist in disaster-affected areas 2. “’Humanitarian assistance’ is aid and action designed to save lives, alleviate suffering and maintain and protect human dignity during and in the aftermath of emergencies […] It is intended to be governed by the principles of humanity, neutrality, impartiality and independence […] and it is supposed to be short-term in nature and provide for activities in the immediate aftermath of a disaster” 7.

Humanitarian assistance amounted to about 9% of the total foreign aid budget in 2011 (12 billion USD) for the government members of the Development Assistance Committee of the Organisation for Economic Co-operation and Development (OECD DAC) 8.

According to agreed principles among major governmental donors of humanitarian assistance, allocations for humanitarian assistance should be distributed based on need and needs assessment (NA) data 9. The Good Humanitarian Donorship is an agreement, established in 2003 by donors, which consist of 23 principles 9. Principle six^1^ of this agreement 9,10 is of particular importance because it operationalizes the humanitarian principles for donors. By making the four above-mentioned principles measurable, it is visible that they can only guide donors when humanitarian assistance is provided in proportion to needs 11. However, criteria for allocation of funds are not always accessible and transparent 3. Therefore, the global humanitarian community has called for improvement of NA in decision-making 12 ^,^ 13.

Several global policy initiatives endorse the use of NA, e.g., the Sphere Project and the Health and Nutrition Tracking Service 12,14. However, these initiatives may not provide sufficient technical support to donors 13,15,16. There is increasing pressure on donors to demonstrate what information they use to justify humanitarian responses 16, such as needs assessments.

Despite the agreed-upon principles, NA is only one aspect that determines how donors allocate humanitarian funds 16 ^,^ 17 ^,^ 18. Bilateral, political, economic, technical and strategic factors, as well as media coverage, also can influence the allocation of funds and provision of humanitarian assistance in disaster situations 2 ^,^ 13 ^,^ 19. In addition there are several challenges with the use of NA. They are often hampered by urgent time frames, security issues and limited access to certain areas 12. Moreover, standardized methods to assess needs have proven difficult to establish 13 and to carry out, especially in rapidly changing context in disaster situations. There are also no commonly accepted indicators that define needs. Needs assessments have been criticized to be of poor quality, often due to limited capacity of humanitarian assistance organizations to conduct assessments 14. Nevertheless, NA remains essential to humanitarian assistance. The Multi-Cluster/Sectoral Initial Rapid Assessment (MIRA) is a, newly established, needs assessment coordination tool by the by the United Nation’s Office for the Coordination of Humanitarian Affairs (OCHA). The MIRA manual addresses some of the above-mentioned constraints. According to OCHA, the MIRA may be used to support donors’ strategic decisions following sudden onset disasters as well as in-depth analyses for each sector 12.

The Swedish International Development and Cooperation Agency (Sida) is a major international donor with a needs-based humanitarian assistance funding policy 10. Sida was one of 16 donors that established the Good Humanitarian Donorship in 2003, with the aim of increasing accountability and effectiveness of humanitarian assistance 9. A study of Sida’s allocation decisions of 2003, highlighted that Sida rarely referred to NA data in decision making 20. In recent years, Sida has continued to develop and improve its needs-based funding strategy and has supported several initiatives to strengthen objective NA, as for example in the Assessment Capacities Project 3. The Development Assistance Research Associates organization yearly evaluates how donor governments’ apply the Good Humanitarian Donorship principles. Among the OECD DAC members, Sweden was ranked 3^rd^ on the Humanitarian Response Index in 2011 21, indicating that it is a well-functioning donor of humanitarian assistance.

The aim of this study is to explore a donor’s use of NA data in decision-making for allocations of funds for health-related humanitarian assistance contributions. To achieve this goal, we assessed to what extent Sida included NA data in assessment memoranda and decision documents for allocation of funds for health-related humanitarian assistance contributions in 2012. In addition, we explored how desk-officers at Sida describe the use of NA data in practice, for the purpose of needs-based allocations.

## Material and Methods

This study design builds on a previously conducted study of Sida 20 with the assumption that *Sida allocates funds based on needs assessment data*. In the current study, some adaptations were made to better meet the aim of this study; thus, a questionnaire was developed (Appendix 1). A mixed-methods design, with both quantitative and qualitative elements, allowed for deep insights into Sida’s use of NA 22. The inquiry strategy was sequentially explanatory, i.e., a first phase of quantitative data collection was followed by a second phase of qualitative data collection 23.

**Definitions**

Generally, there are two main types of disasters, natural and man-made; the latter may develop into complex emergencies 5,24. Disasters may be categorized as sudden and slow onset disasters 24.

For the purpose of this study, needs assessment (NA) was defined as “a systematic collection of information on the magnitude of the disaster and the human needs to be addressed” 18. This information enables an analysis of response requirements 25.

Health related contributions were identified based on the following criteria: contributions targeting the *accessibility* of preventive and curative health and psychosocial services; contributions targeting the *performance* of health services, epidemic surveillance and/or coordination of health-activities; and contributions by water and sanitation components and/or nutrition components. 17 ^,^ 26

**Setting**

In 2012, Sida’s humanitarian assistance budget amounted to 2 838 million SEK^2^ (410 million USD) 27, accounting for 12.7 % of the official development assistance 21. However, the total sum of Swedish humanitarian assistance amounted to about 5 000 million SEK, as the Ministry for Foreign Affairs was responsible for un-earmarked base budgets that went directly to multilateral organizations, mainly within the UN-system. Allocations to on-going crises amounted to 70% (1 848 million SEK) of the total budget; of which five multi-annual framework agreements received 21%. Furthermore, 25% (660 million SEK) was allocated for sudden onset disasters (SOD) or deterioration of on-going crises. Eight partner organizations had Rapid Response Mechanism agreements with Sida (300 million SEK), receiving pre-positioned funds annually 28. The number of humanitarian assistance contributions has decreased; in 2011 Sida distributed funds to 113 contributions compared to 50 contributions in 2012 27.

The allocation process follows different steps for on-going crises compared to sudden onset disasters. In brief, Sida has established 11 criteria that are used yearly to identify how to allocate between crises on a global level, such as for global needs assessment of the European Community Humanitarian Office and for the forgotten crises assessment as well as the Consolidated Appeal Process (CAP) by the United Nation’s Office for the Coordination of Humanitarian Affairs 28. The European Community Humanitarian Office provides global needs assessment and forgotten crises assessment on a yearly basis, by combining vulnerability and crises indices 29. Based on identified geographic areas, Sida compiles its Humanitarian Country Analyses to identify how to allocate within a given crisis as parallel to organizational assessments, compiled by desk officers 28. Sida used three main strategies in 2012 to enable rapid responses following sudden onset disasters. The donor initiated these following a flash appeal by the United Nation’s Office for the Coordination of Humanitarian Affairs or an emergency appeal by the International Committee of the Red Cross. Sida then approved Rapid Response Mechanisms, provided funds to Emergency Relief Funds or added funds to previous agreements with partner organizations 28.

**Document Analysis**

We reviewed all Sida funded humanitarian assistance contributions in 2012. These included *assessment memoranda* (‘Bedömningspromemoria’) and *decision documents* (‘Beslut om insats’). We also reviewed documents on *appraisal of intervention*, when assessment memoranda were absent, which was the case for 12 contributions. The size of the documents ranged from one to 28 pages. Documents were acquired electronically from a program administrator at Sida. Documentation concerning Sida allocations is open government data. These official Swedish documents are thus publicly available 30.

Content was analyzed to identify and quantify NA data in a systematic manner through descriptive statistics. To measure quantified health needs, we used a pre-determined checklist developed by von Schreeb et al., which had previously been tested on similar documents for the same purpose 20. The checklist allowed us to identify sectors and sizes of humanitarian assistance contributions as well as actors. Additional key components were humanitarian health related indicators e.g., mortality, malnutrition data, and access to health care, access to water, and water quality and quantity, as well as the size of the target population and cost per capita.

Data were organized by contribution by coding indicators on NA data as True or False. Subsequently, frequencies of needs assessments were identified by cross tabulation and summarized in Excel. The total budget used for calculations was 2640 million SEK, without the post specific support for conflict affected areas (‘konfliktanslaget’) 28.

The Rapid Response Mechanisms contributions were not analyzed further, because no decision documents were available after Sida signed and approved the requests documents (‘Hemställan’) from humanitarian assistance organizations. More than 200 Rapid Response Mechanisms requests were approved by Sida in this manner 27.

**Key Informant Interviews**

To select informants, five people who compiled assessment memoranda for health-related contributions in 2012 were selected. Three of them expressed interest in participating. The informants recommended additional staff members to interview. Thus two people, who were part of the decision-making process of 2012 and/or 2013, were also invited to participate. One additional staff member was in turn invited by one of these informants. Taken together, a total of six people out of 20 at the Humanitarian Unit of the Department for Conflict and Post-conflict Cooperation at Sida were interviewed.

The interview guide was semi-structured with a range of set topics that served as a foundation, allowing the interviewee to decide what information s/he felt comfortable communicating and its relative importance for each of the topics 22. Interviews, which lasted 30-60 minutes, were conducted in Swedish and audio recorded following consent by the interviewee. Interviews took place at Sida’s head office in Stockholm in April and May 2013.

The interview data was analyzed by thematic content analysis 22. Interviews were transcribed verbatim and the transcriptions were subsequently condensed to contain information that carried meaning on a manifest level 31, related to the second objective of this study. We did not attempt to interpret any latent meanings in the data from the recordings nor from the transcriptions. Interviewees were given the opportunity to comment on an English summary of their interview. Moreover, selected quotes were translated from Swedish to English. The interview guide (Appendix 1), with three overarching themes, served as a foundation for the analysis. Segments, sentences or phrases identified with color codes were grouped into categories and summarized 22.

## Results

**Document Analysis**

Out of Sida’s 50 contributions to humanitarian assistance seven were excluded because they contained classified information, were amendments of agreements, or of administrative nature. A further 18 contributions were excluded because they lacked health care, water and sanitation components, and/or nutrition components (Figure 1).

**Sida’s allocations in 2012 for humanitarian health assistance contributions 2640 million SEK:**
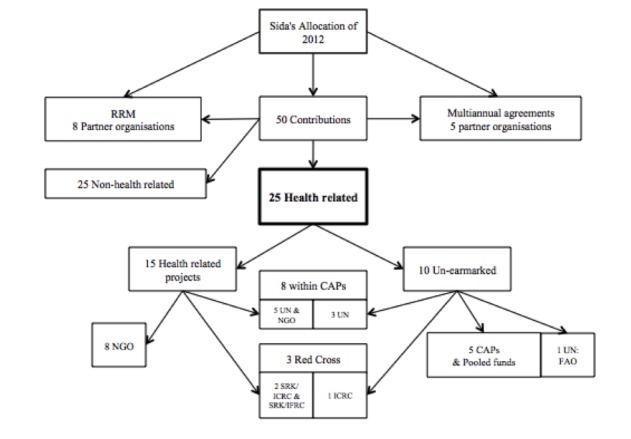
RRM: Rapid Response Mechanism, NGO: Non-Governmental Organization, CAP: Consolidated Appeal Process, FAO: Food and Agricultural Organization of the UN, SRK: Swedish Red Cross

The remaining 25 contributions were health-related and were included in our analysis (Figure 1). Out of these, 15 contributions contained health care components, 20 had water and sanitation components and 17 had food and/or nutrition components. Several health-related contributions had more than one sector component, and several contained a number of projects in different crises. For ten health-related contributions Sida delegated responsibility to another humanitarian assistance organizations to decide on further allocations. The remaining 15 contributions were earmarked or not further delegated in the assessment memoranda and/or in the decision documents (Figure 1).

Sida’s budget of health-related contributions amounted to 1 544 million SEK (58% of the total budget for humanitarian assistance). The majority of Sida’s allocations, 1 074 million SEK, were un-earmarked health related contributions (Figure 2). Four contributions amounted to more than 100 million SEK, with a combined amount of 991 million SEK (38% of the total budget for humanitarian assistance). The two largest allocations were distributed to the International Committee of the Red Cross and the Swedish Red Cross, which respectively received 450 million SEK and 208 million SEK.

**Sida’s budget allocation for health related humanitarian contributions in 2012 in million Swedish crowns (million SEK).**
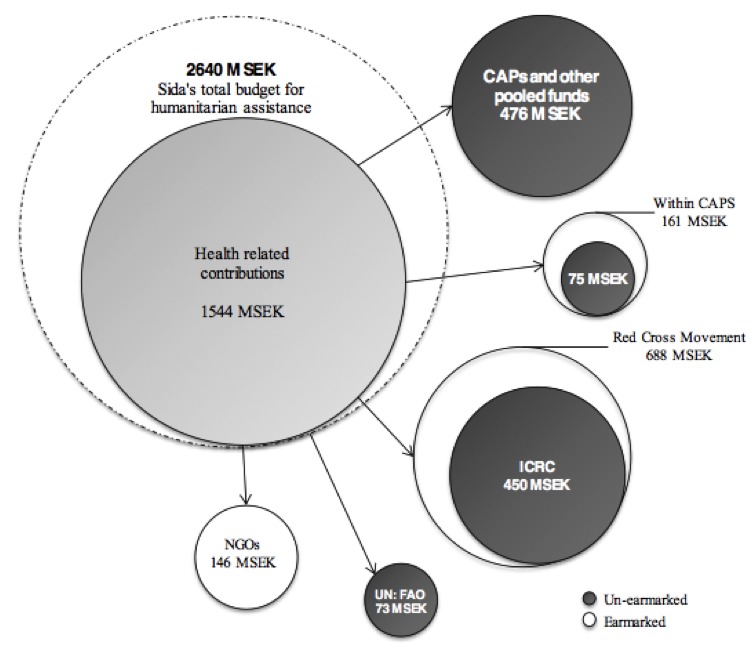

In 92% of the contribution cases, data on the total population size in the project area was absent (Table 1). Reference to the size of the target population was available in 14 out of the 25 reviewed assessment memoranda and decision documents. Sida included estimates of cost per capita in five of the 25 contributions (20% of the reviewed documents).

Access to health care was referred to in eight of 25 (32%) of the decisions. Information on whether a specific amount was allocated for a health care project within a contribution was found in one document. Inclusion of quantified health needs was identified in 42% of the documents.

**Table 1. Humanitarian health related contributions with NA data in assessment memoranda and decision documents:** 

Indicator	Decisions with data	n	%
Populations size (all contributions)	2	25	8%
Mortality (health care component)	5	15	33%
Malnutrition (food and nutrition component)	8	17	47%
Water quantity and/or quality (WASH components)	11	20	55%

More NA data was available in un-earmarked assessment memoranda and decision documents. In these records, data on mortality differed between 13 to 30%, while malnutrition ranged from 27 to 50% and for contributions with water and sanitation components NA data ranged from 47 to 50%. The calculated mean sum for un-earmarked contributions was 107 million SEK. In contrast, the mean sum for the remainder, mainly funds to NGOs, was 31 million SEK.

**Key Informant Interviews**

**Notion of Needs Assessment**

Informants defined needs assessments (NA) in various ways. Their descriptions proposed four different elements: the principles guiding humanitarian assistance, the methods of data collection, and the different levels of NA in the allocation process as well as the components that should be included. One informant stated that, *“There is no shared definition of needs assessments”*.

Taken together, the informants described that Sida uses NA on three different levels: globally, regionally and on a national level. The most important components were suggested context dependent yet ranged from information on specific indicators such as maternal mortality rates to defining the most important component as the Consolidated Appeal Process (CAP). One informant explained,*“There will never be a time when we have strictly scientific and objective needs assessments”.*

**Allocation ‘According to Need’**

All informants highlighted the importance of NA as a foundation for allocations. They further described the allocation process as complex; assessing Sida’s use of NA for on-going crises should start from the donor’s global NA and result in decision documents for contributions. A majority of the informants described NA to be one essential criterion, out of the eleven criteria (Appendix II) established for input values for allocation between on-going crises. The weight of the different criteria was described as context-dependent, hence the most important one was not possible to establish. The informants jointly summarized Sida’s global allocation for 2013 as needs based. Yet, according to one informant, the initial stage of Sida’s allocation process was mainly an internal process. While another informant argued, *“I believe that our global needs assessment is more fair today than before”.*

Collectively, the informants expressed that weighing needs is not a straightforward practice, nor is it clear how to assess where the most pressing needs are geographically. One informant brought up the definition of ‘need’ as an issue complicating the allocation according to need. The aspired needs-based allocation is influenced by how *“pure” *Sida wishes the allocation to be, which means that it is dependent on the definition of need and *“if we choose to see the needs or not”*.

**Available Information and Quality of the Material**

A majority of the informants agreed that the vast amount of available information meets the type of information requested by Sida. A widely held view among the informants was that the lack of reliable data is a main problem. Several informants emphasized that NA should be updated and verified on a regular basis, *“what you write in October is obsolete in December”*.

All the informants articulated that the Consolidated Appeal Process constitute an essential foundation for needs-based allocations, yet they expressed awareness that the numbers presented the CAP documents are neither trustworthy nor comparable. Sida uses CAPs to assess needs both globally and sector specifically; consequently, if a country lacks a CAP, an important piece of information is lost. *“Hence we make large decisions on several hundred million [Swedish] crowns on information that we do not know for sure is correct”*. Yet it was stated that Sida has judged available information, despite it being “*inadequate on all areas*”, to have a level of trustworthiness high enough for bearing decisions.

Data from villages where humanitarian organizations have access may not provide an accurate picture of a whole crisis. Information may come from informal sources, such as from journalists, when formal NA is hampered in restricted areas *“Exactly what the situation looks like is unknown. All we know is that there are great needs”.* In addition, one informant suggested that trends influence narrow data collection on certain topics as providing this type of data will grant funds and thus *“the need is overestimated”*. Another interviewee indicated that data that organizations select to present in applications to Sida is biased.

**Validation of Data**

The informants stated that there was no systematic method of validating information, *“there is no documented method”*. One informant mentioned that experience gained in a certain sector in the field might influence interpretation of data and what s/he spontaneously looks for in an application. In addition, the difficulties in knowing what benchmarks to set when assessing the quality of NA were brought up. Three informants mentioned the phrase *“good enough”* in terms of assessing quality of data, in addition to *“* *making an assessment of what is reasonable”*. Due to different time frames, one interviewee described that the process of monitoring data looks different following Rapid Response Mechanisms compared to on-going crises.

One informant expressed the need for the United Nation’s Office for the Coordination of Humanitarian Affairs to take on a more independent role including validating data, because Sida does not find this task appropriate for donors. A majority of the informants stated that they crosscheck information by consulting embassy and field personnel, as well as Sida personnel with geographic expertise. Sida also receives external support with NA from the Assessment Capacities Project and from an external help-desk. Moreover, one interviewee argued that the Consolidated Appeal Processes often stem out of politicized processes, linked to vested interests based on mandates. Another informant thought that an active approach in CAP workshops is a way of validating the numbers and judge political overtones.*“With our presence as a donor in these forums we can feel and assess, make our own assessments”*.

**Influence of Other Factors on the Link between NA to Decisions**

The informants reported a number of factors limiting good NA; sometimes concurrent negative factors such as access and corruption.* “If you cannot gain access [to an area] you cannot conduct a proper needs assessment. It’s a blank spot”. *Sida has enabled quick assistance to these areas once there is access, by allocations to un-earmarked humanitarian funds. Sida must also relate to the security status of an area, vulnerabilities, as well as time and capacity to avoid misguided allocations, something that a majority of the interviewees indicated.

Allocation can either be increased or decreased depending on whether a crisis is categorized as forgotten, has access problems, or there is reason to believe that the Consolidated Appeal Processes (CAPs) are inflated. In addition, Sida explores its comparative advantages and in the event of donor fatigue, a disproportionately large allocation may be appropriate. Cost per beneficiary, specified in CAPs is considered; one informant highlighted the value of this information for cost-effective allocations and to assess fund-raising aspects.

Sida conducts overall assessments of implementing organizations by assessing their capacities and priorities as well as their previous experience in areas identified in the global NA. The informants emphasized the importance of Sida to build up a high level of trust with its partner organizations and rely on their ability to conduct NA.

**Response following a Sudden Onset Disasters**

Following an SOD, Sida prioritizes quick**responses with its Rapid Response Mechanism (RRM). Through this mechanism, Sida assesses the implementing organization at an early stage and evaluate the capacity, priorities of the organization and its capability to identify and respond to needs when they occur. Hence, the RRM request sent to Sida should contain information on the specific needs in regards to the specific context. *“However, behind this quick response […] when it comes to rapidly analyze relevance and needs, there is a comprehensive assessment making an organization an RRM-partner”.*

**Value of NA versus its Pragmatic Use**

Several of the interviewees described that specific information, when available, is valuable since it constitutes the foundation for Sida’s needs-based allocations, subsequently reaching the ones with the most pressing needs. One informant discussed the aspect of valuing secondary information higher, as *“The Achilles’ heel for needs assessments is that a well-written report is composed and it becomes obsolete”.* Sida’s use of NA was further described as challenged due to a *“system error” *in the humanitarian sector due to a lack of independent actors.

The informants further described difficulties in knowing which indicators to look for in a given context as well as the lack of meta-analyses. Two informants expressed a wish to see all organizations presenting NA data in a consistent manner to enable comparisons. As one informant explained, *“There will always be a tension between having some standardized model and something that will be fair for all countries, all organizations and everyone affected by the crisis and at the same time be flexible and adapt to the context”. *However, there were different thoughts on this impression as one informant expressed the wish to continue using NA* “the way we do”.*

## Discussion

The findings of our document analysis showed that Sida had a clear and well understood decision-making process. However, in the reviewed assessment memoranda and decision documents, Sida made no systematic reference to needs assessment data. Considering the needs-based funding policy, few of the reviewed documents contained NA data. The extent of which NA was included in the documents does not illustrate to what extent it informed funding decisions; as the interviews indicated that other factors need to be taken into account as well in order to avoid misguided allocations.

More NA data was found included in the documents for un-earmarked contributions. However, it cannot be determined whether this was due to the link between inclusion of NA data and size of the contributions. An alternative explanation may be that global indicators are included to a greater extent in the Consolidated Appeal Processes and similarly pooled funds. Yet information found in these documents may be too comprehensive to enable an analysis of response requirements for donors.

We found that the initial phase of the allocation process was mainly internal and learned that assessment memoranda and decision documents were insufficient to understand a needs-based decision. Based on these observations, the transparency of Sida’s use of NA in the allocation process could be improved. In 2013 Sida has adapted its method to allocate humanitarian funding for on-going crises, the results of this remain to be determined.

Compared to the 2003 Sida study, our findings indicate an improvement in making reference to NA data in decisions for funding. The inclusion of mortality rates increased from 4 to 33%, those for malnutrition from 11 to 47%, and those for water quantity and/or quality from 16 to 55% 20. This increase may also be due to more NA data being collected and available in 2012 compared to in 2003.

Since 2003, Sida has established a selection of strategic partner organizations. A large proportion of the allocation was directed to/or within the Consolidated Appeal Processes and the Red Cross movement. Sida’s budget for humanitarian assistance grew by 70% between 2003-2012 (1546 to 2640 million SEK) 20. For the same time period, we identified a decrease in the number of contributions. In 2012, 50 contributions were funded compare to 204 in 2003 20. One explanation for this decrease may be that Sida’s yearly global NA results in a more planned allocation process. In 2003 more single project applications received funds 20.

Similar to the findings a decade ago, Sida still considers it relevant to assess the implementing capacity of the organization requesting funds. As well, Swedish Embassies and field personnel continue to be an important supplement to NA data 20. Yet Sida’s new contribution system for on-going crises is a step towards a more global proportionate allocation with increased transparency in the decision-making process.

Neither Sida’s nor the Good Humanitarian Donorship policies for needs-based allocations have changed over the past decade. The low inclusion of NA data in the reviewed documents may be the result of an ambiguous policy. The policy needs to be rewritten or better clarified if its purpose is to guide donors and enable measurement of their performance. In its current interpretation, personal opinions of those deciding about funding influence decisions. A clear and widely accepted definition of what constitutes a NA, what indicators decision makers should build on, and how results may be gauged and compared, is needed. In order to translate needs into funding additional factors such as risk and vulnerabilities need to be taken into account and included in an allocation formula. We are not aware of the existence of such formula among any donor.

Our findings should not be interpreted as a sign of bad adherence to policies. Sida is a well-functioning humanitarian donor. Instead, our findings indicate that needs-based allocations are not a straightforward process. The concept of NA as a driver for funding is appealing and intuitive but may, according to the study results be unrealistic to achieve. The purpose of NA is not only to serve as basis for funding but also to direct humanitarian assistance projects and provide baseline data. It may not be realistic that the same NA data should serve so many purposes. Perhaps donors should use other mechanisms to guide funding. For example, Sida allocated a majority of the health-related contributions to un-earmarked funds, which illustrates the flexibility of the donor. To that end, it is noteworthy that four contributions accounted for 38% of Sida’s total budget for humanitarian assistance, and three of these were un-earmarked. Consequently, Sida allowed other actors in the humanitarian arena to decide on further distributions. Such flexibility in approach may be beneficial, especially given that agencies in the field are well suited to identify needs. Yet it may be contested if it is appropriate that the implementer defines the needs that should be addressed.

Sida pre-positioned 25% of its budget in 2012 to enable rapid responses in the event of sudden onset disasters (SOD) or sudden deterioration of on-going crisis. This mechanism seems rational given the context; yet, the allocation is not in accordance with principle six of the Good Humanitarian Donorship. Assessing needs following an SOD is very different from monitoring needs in a protracted complex emergency. In the first case, assumptions building on empirical evidence from previous disasters in combination with vulnerability data and the magnitude of the SOD will help estimate the needs. In the latter case, systematic monitoring and using well-established indicators will help determine severity and needs. Thus, NA is not a generic activity that can be launched in any context.

Our findings correspond to previous publications suggesting that factors other than NA may influence decision-making for humanitarian assistance 13 ^,^ 16. Despite the fact that the Consolidated Appeal Process documents have improved over the past years, the informants described the lack of reliable information as a main problem for the entire humanitarian sector. Sida, like other donors 32, relies on data from CAPs. Yet the credibility of data in these documents has previously been described as weak and arbitrary 16. Moreover, the complexity of defining needs is a central problem for the whole humanitarian sector 11. Although Sida’s global NA was described as essential in order to establish needs-based allocations for on-going crises, ambiguous concepts complicate the interpretation of a proportional global distribution. It has previously been argued that this ambiguity results in inconsistent humanitarian action 13.

It is important to highlight the reliability of studying assessment memoranda and decision documents as the point in the allocation process to assess Sida’s use of NA data as a basis for decisions. These documents may not be representative of the extent to which NA data was used for needs-based decisions. It is possible that Sida considered NA data at an earlier stage of the allocation process.

The interviews reinforced and compensated for limitations in the document analysis (23), although the interviewees should not be considered representative of Sida as an organization. Purposive sampling of informants may also affect the credibility of the results. In addition, the definitions used for this study may have influenced the design of the interview guide and our interpretation of the results.

Sida has taken an active role in continuously improving the use of NA data 3. Nevertheless, incentives for humanitarian assistance organizations to improve NA data may be harmed if one of the highest ranked donors by the Development Assistance Research Associates accepts inadequate data such as CAPs. It appears that the entire humanitarian community operates with inadequate NA 13 ^,^ 16 ^,^ 19. Thus, it should be the collective responsibility for the humanitarian community to improve objective assessments 9. A scientific and objective evaluation of needs may not be attainable within this context. As no generally agreed upon systematic methods of assessing needs exist, further research may address whether vulnerabilities of population groups and severity of disasters, in addition to needs, would provide a more objective basis for decisions on funding allocations for donors.

## Conclusions

The fact that Sida, which is a credible and well-organized donor of humanitarian assistance, did not systematically refer to NA data in its funding decisions raises questions. This lack of reference cannot be explained by institutional incapacity of Sida, a well-functioning and respected humanitarian donor that has received high rankings in evaluations 21. Although it is possible that funding based on NA data is an unrealistic endeavor, it is likely that absence of NA in decision-making may be due to a gap between policy and practice.

As a first step to allow realistic needs-based funding, defined indicators that capture human needs must be determined, specified, and widely accepted. The next step toward this goal is to develop a framework that interprets the severity of the indicators. Such a step will also allow trend analyses and comparisons between crises. Donors should take the lead in developing such a framework to give justification to their policy that is presently difficult, if not impossible to evaluate.

Overall, did Sida do well in funding based on needs assessment data? The answer is that we do not know. To determine what ought to be the benchmark level for funding based on NA data, more studies from other donors are needed to move the process of needs-based funding forward. Currently, great efforts are in place for collecting NA data, but how this information should be used and interpreted needs much more attention.

## Footnotes

^1^ “Allocate humanitarian funding in proportion to needs and on the basis of needs assessments”

^2^ 6.92 SEK = 1 USD 1st January 2012

## Appendix 1

### Interview guide [translated from Swedish]

**PART 1, Definition of the concept needs assessments**

1. How would you define needs assessments?

-       Can you provide examples of the most important components?

2. Can you tell me about your role in the allocation process?

3. How do you use needs assessments when compiling an assessment memorandum?

-       How will Sida’s new allocation process affect the use of needs assessments?

4. How would you describe and define ’allocate according to need’, according to established guidelines?

**PART 2, Available information and material**

1. What type of needs assessment do you find useful?

-       Which are the most important sources?

-       Can you provide an example on how the use of information differs in different contexts?

2. Does available information meet the kind you request?

-       Is baseline or disaster-specific information asked for?

-       Are relative or absolute measurements of need asked for?

3. Which methods do you use to validate needs assessments?

-       Do you look for something in particular when assessing the quality of a needs assessment?

-       What is the consequence of inadequate information?

-       How is an inadequate needs assessment compensated for?

**PART 3, Other factors’ influence on decisions**

1. What other factors would you consider influencing a decision?

-       What is the consequence of decisions taken for Rapid Response Mechanism- contributions in the absence of assessment memoranda?

2. How would you like to use needs assessments?

-       What is the value of having specific information available?

-       What does needs assessment contribute to in an assessment memorandum?

**FINAL**

Can you estimate to what extent decisions are based on needs assessments?

Lastly, is there anything you would like to add?

## Appendix 2

### Sida's method to allocate humanitarian funding across large on-going crises in 2013

**“1. ECHO Global Needs Assessment (GNA) and Forgotten Crises Assessment (FCA)**

- ECHO’s GNA on vulnerability and crises, which categorizes countries on the basis of existence of crisis (manmade or natural disaster) and degree of vulnerability on a scale of 1-3 (with 3 signifying the most vulnerable and crisis-affected).

- ECHO’s GNA-rank joins the vulnerability and crises indexes into a global ranking (with 1 signifying the most vulnerable and crisis-affected).

- ECHO’s Forgotten Crises Assessment, which identifies crises that have been overlooked or neglected by the international humanitarian community and/or the global media which need special attention.

**2. Number of affected people**

- Number of affected people in a crisis, based on information contained in CAP or flash-appeals, has been compared with total number of affected people in the 22 countries […]

- Proportion of affected people has been compared to the proportion of Sida funding.

- Cost per beneficiary in CAP compared to the cost in other crises.

**3. Key funding facts**

- Size and funding level of CAPs in 2012

- Increase/ decrease in the need/size of the CAP between 2012 and 13.

**4. Other criteria**

- Sweden’s role as a humanitarian coordinator

- Humanitarian access

- Opportunities for following-up humanitarian assistance

-Links to Swedish development cooperation” (33) 
